# AIDS patients have increased surfactant protein D but normal mannose binding lectin levels in lung fluid

**DOI:** 10.1186/1465-9921-8-42

**Published:** 2007-06-13

**Authors:** Kondwani C Jambo, Neil French, Ed Zijlstra, Stephen B Gordon

**Affiliations:** 1 Malawi-Liverpool-Wellcome Trust Clinical Research Programme, Blantyre, Malawi; 2 Wellcome Trust/LEPRA Karonga Prevention Study, London School of Hygiene and Tropical Medicine, Chilumba, Malawi; 3 Department of Medicine, University of Malawi College of Medicine, Blantyre, Malawi; 4 Liverpool School of Tropical Medicine, Liverpool, UK

## Abstract

**Background:**

Surfactant protein D (SP-D) and Mannose Binding Lectin (MBL) are collectins that have opsonic and immunoregulatory functions, are found in lung fluid and interact with the human immunodeficiency virus (HIV). We compared collectin levels in lung fluid and serum from HIV infected and normal subjects to determine if alterations in lung collectin levels were associated with HIV infection and might result in increased susceptibility to other pulmonary infections.

**Methods:**

Blood and bronchoalveolar lavage samples were collected from 19 HIV-infected individuals and 17 HIV-uninfected individuals, all with normal chest X ray at time of study. HIV viral loads and peripheral blood CD4+ T cell counts were measured in all subjects. SP-D was measured in lung fluid, and MBL in both lung fluid and serum.

**Results:**

SP-D levels were not significantly different in lung fluid from HIV-uninfected (median 406.72 ng/ml) and HIV-infected individuals with high CD4 count (CD4 >200) (median 382.60 ng/ml) but were elevated in HIV-infected individuals with low CD4 count (median 577.79 ng/ml; Kruskall Wallis p < 0.05). MBL levels in serum were not significantly different between HIV-uninfected and HIV-infected individuals (median 1782.70 ng/ml vs 2639.73 ng/ml) and were not detectable in lung fluid.

**Conclusion:**

SP-D levels are increased in lung fluid from AIDS patients but not in patients with early HIV infection. MBL levels are not altered by HIV infection or AIDS. There is no evidence that altered pulmonary collectin levels result in susceptibility to infection in these patients.

## Background

Surfactant protein D (SP-D) and mannose binding lectin (MBL) are members of the human collectin system. The collectins are a group of molecules characterised by a collagenous region and a lectin (carbohydrate-binding) domain which together give the members structural and functional similarity[1]. The collectins function in innate immunity as opsonins and agglutinins but also have important pro- and anti-inflammatory immunomodulatory functions[2]. Surfactant protein D is produced mainly in the lung by alveolar type II cells and bronchiolar epithelial cells, but has also been reported at other mucosal surfaces[3]. Mannose binding lectin is produced in the liver as an acute phase protein which may leak from the systemic circulation at the site of inflammation in the lungs, and provide an optional host defence mechanism[4].

Alterations in collectin levels are associated with susceptibility to pulmonary infection. Levels of SP-D are decreased in bronchoalveolar lavage fluid of cystic fibrosis patients and relative collectin deficiency is inversely related to inflammation in these patients[5]. SP-D is critical in modulating responses to respiratory viral infections[6] and bacterial pneumonia[7]. It is present in serum of healthy adults ranging from 158 – 3711 ng/ml but SP-D in serum is considered to reflect damage to or response from epithelial cells to inflammation[7]. Reduced levels of MBL in serum are associated with meningococcal invasion through the respiratory tract[8,9] and MBL was recently shown to be a critical determinant of macrophage ingestion of meningococci[10]. MBL was present in bronchoalveolar lavage samples from patients with pneumonia at concentrations ranging from 0.011 to 0.078 mg/ml but none was found in bronchoalveolar lavage from healthy adults[11].

SP-D and MBL play an important role in defence against HIV infection. SP-D binds to the HIV surface protein gp120 and has significant HIV-binding and inhibitory activities[3]. SP-D expression has recently been measured in both respiratory and non-respiratory mucosa including the oral cavity and female genital tract[12,13] suggesting a possible role in sexual or vertical transmission of HIV. SP-D inhibits HIV infectivity at significantly lower concentrations than MBL[3]. Increased susceptibility to HIV infection in patients with MBL insufficiency or protection from HIV in those with high MBL levels has been reported[14,15] but the effect of reduced levels of MBL on HIV disease progression are controversial[16,17]. MBL initiates complement activation[18] and can also inhibit DC-SIGN-mediated transfer of HIV from dendritic cells to T cells[19].

Our hypothesis was that reduced levels of collectins in BAL might result in increased susceptibility to pneumonia among AIDS patients. Our goal in this study was to determine if HIV status (stratified by CD4 count) was associated with altered levels of collectins in BAL and serum.

## Methods

### Subject recruitment and sample collection

Adult Malawians were recruited by advertisement and gave written informed consent to participate in a study of pulmonary immune responses to infection. This study included bronchoscopy with lavage, serum sampling and HIV testing. This study was approved by the Liverpool School of Tropical Medicine Research Ethics Committee and the College of Medicine Research Ethics Committee of the University of Malawi.

Patients attended recruitment clinic when venous blood was collected. Bronchoscopy with lavage was carried out a few days later as previously described[20]. Briefly, a fibreoptic bronchoscope was wedged in a sub-segmental bronchus of the right middle lobe and 200 ml of warmed sterile saline introduced in 4 aliquots. Bronchoalveolar lavage (BAL) obtained by this method typically yields 120 ml of cellular fluid. BAL and venous blood samples were transferred on ice immediately to the laboratory and centrifuged to remove the cellular pellet. Supernatant fluid and serum obtained from venous blood were stored at -80°C for future assay.

### Laboratory assays

Serum HIV viral loads and CD4+ T cell counts were determined by Amplicor HIV-1 Monitor Test version 5.0 and Becton Dickinson FACS Count, respectively.

Measurement of SP-D in BAL was done using an SP-D Sandwich ELISA kit (BioVender GmbH, Germany). The Standards and Quality Controls used in this kit are both human recombinant protein based. The Assay was done in accordance with the manufacturer's instructions.

Measurement of MBL in serum and BAL was done using an MBL ELISA kit (Sanquin, Netherlands). The kit has a minimum detection level of 9.0 ng/mL and a measurable concentration range of 9.0 to 350 ng/mL. The Assay was done in accordance with the manufacturer's instructions.

### Statistical Analysis

HIV-infected patients were stratified into two groups according to peripheral blood CD4+ T lymphocyte cell count greater or less than 200 cells/ml. This corresponds to a clinical diagnosis of AIDS. We compared levels of SP-D and MBL in BAL and serum by HIV status using Mann Whitney test and Kriskall Wallis test. The results were reported as median with interquartile ranges. Intercooled Stata 9.2 was used to perform all the statistical operations in this study. All the graphs in this study were produced using GraphPad Prism 5.00.

## Results

### Subjects

Blood and BAL samples were collected with informed consent from 19 HIV-infected and 17 HIV-uninfected individuals, all of whom were healthy at the time of bronchoscopy and had a normal chest X ray. The demographic and clinical characteristics of the three groups are summarised in Table 1. There was no significant difference in age distribution between the groups. There was a higher mean HIV viral load in both the serum and BAL of subjects with CD4 counts less than 200 cells/μl (clinical AIDS) than in HIV infected subjects with higher CD4 counts. None of the HIV infected subjects was on anti-retroviral therapy at the time of the study. Three subjects with AIDS were current cigarette smokers of 3, 5 and 20 cigarettes per day (1.5, 5 and 15 pack-years) and two normal subjects were current smokers of 5 and 6 cigarettes per day (both 3 pack-years). One HIV infected subject with a normal CD4 count (greater than 500 cells/μl) had stopped smoking in 1971 (1.5 pack-years).

**Table 1:** Demographic and clinical features of subjects in the study

**Clinical feature**	**Normal**	**HIV infected (CD4 >200 cells/ul)**	**AIDS patients (CD4<200 cells/ul)**
Gender M:F	17 : 2	8 : 2	6 : 1
Age, Mean (± SD), years	30 (9.9)	33.6 (9.4)	34.5 (7.2)
CD4+ T cell count, mean (range), cells/μl	606 (512–978)	493 (368–675)	149 (46–179)
*Plasma *HIV-1 load, mean (range) copies/ml	0	1.4 × 10^5 ^(0.008–4.0 × 10^5^)	2.3 × 10^5 ^(0.3–6.6 ×10^5^)
*BAL Fluid *HIV-1 load, mean (range) copies/ml	0	129 (0–670)	175(0–550)

Abbreviations: BAL = Bronchoalveolar Lavage, SD = Standard Deviation

### Surfactant Protein D in lung fluid during HIV infection

**Figure 1:**
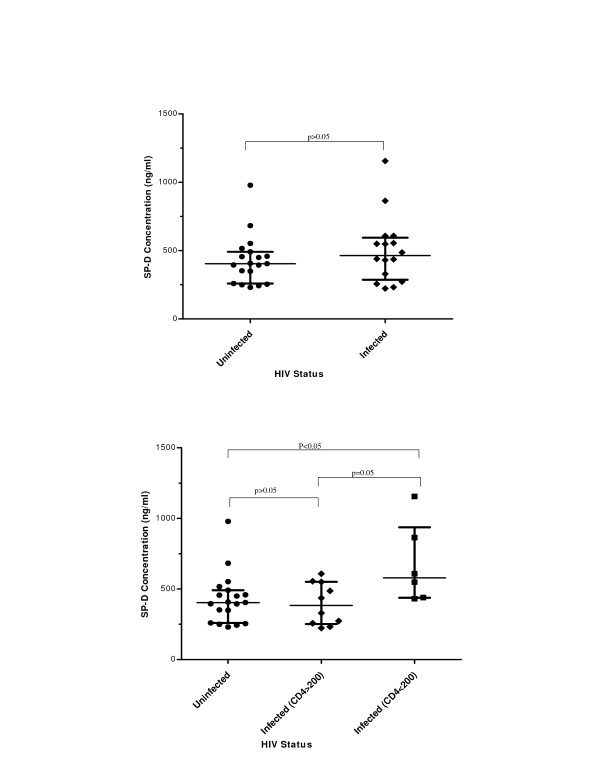
**Levels of SP-D in BAL of both HIV-uninfected and HIV positive individuals**. The bars represent median and interquartile range. **a)**. The analysis was performed on 19 HIV-uninfected and 16 HIV Positive subjects. **b)**. The analysis was performed on 19 HIV-uninfected, 10 HIV Positive with CD4 count > 200 and 6 AIDS patients (CD4 count < 200).

The levels of SP-D in BAL of HIV positive individuals were compared with levels of SP-D in BAL of HIV-uninfected individuals. These levels were not significantly different with median SP-D in HIV uninfected 406.72 ng/ml compared to 463.05 in HIV infected (Mann Whitney p = 0.32) (Figure 1a). We also compared the levels of SP-D among normal subjects, HIV positive subjects with CD4 count >200 cells/μl and patients with AIDS (CD4 count <200 cells/μl). SP-D levels were significantly higher in AIDS patients (median 577.79 ng/ml) compared to normal subjects (median 406.72 ng/ml; Kruskall Wallis p = 0.03) or HIV-infected individuals with CD4 count greater than 200 cells/μl (median 382.60 ng/ml; Kruskall Wallis p = 0.05) (Figure 1b).

As it has been reported that SP-D has significant HIV-binding and inhibitory activities exceeding MBL[12], we investigated the possibility that high levels of SP-D were associated with low viral loads in BAL. SP-D levels were found to have a weak relationship with BAL viral loads (p < 0.05, r^2 ^= 0.26). We also compared SP-D concentration to CD4 count, it was found that there was a weak relationship between the two variables (p < 0.05, r^2 ^= 0.30)

### Mannose Binding Lectin in serum and BAL during HIV infection

**Figure 2:**
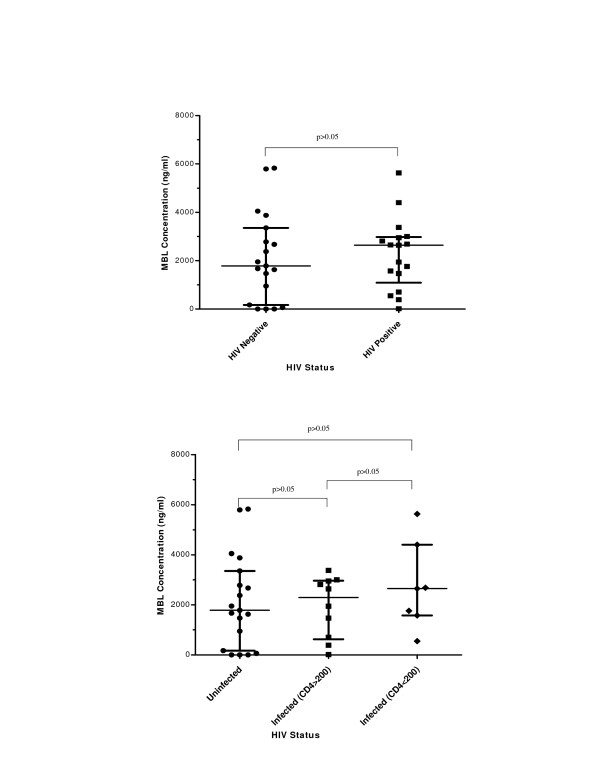
**Levels of MBL in serum of both HIV-uninfected and HIV positive individuals**. The bars represent median and interquartile range. **a)**. The analysis was performed on 19 HIV-uninfected and 17 HIV Positive subjects. **b)**. The analysis was performed on 19 HIV-uninfected, 10 HIV Positive with CD4 count > 200 and 7 AIDS patients (CD4 count < 200).

MBL levels in serum compared by HIV status were not significantly different as shown in Figure 2a (median MBL in normal 1782.70 ng/ml compared to 2639.73 ng/ml in HIV infected; Mann Whitney p = 0.58). We also compared the levels of MBL among normal subjects, HIV positive subjects with CD4 count > 200 and AIDS patients (CD4 count <200 cells/μl). MBL levels were not significantly different among these three groups as shown in Figure 2b (median MBL in normals 1782.70 ng/ml vs HIV positive individuals (CD4 count >200 cells/μl) 2291.73 ng/ml vs AIDS patients 2651.74 ng/ml; Kruskall Wallis p = 0.70).

MBL levels in BAL were below the lower limit of detection in all samples. Using extrapolated data, MBL levels in BAL compared by HIV status showed no significant difference (median MBL in normal 0.283 ng/ml compared to 0.401 ng/ml in HIV infected; Mann Whitney p = 0.06). MBL levels in BAL showed a significant correlation with MBL levels in serum in both HIV-infected and HIV-uninfected individuals (p < 0.05). MBL levels in serum or BAL in HIV infected patients did not show significant correlation with CD4 count or HIV viral load in either plasma or BAL (p > 0.05).

## Discussion

In this study, levels of SP-D and MBL in BAL and serum collected from HIV infected patients were not different from those in normal subjects. Among HIV infected patients, patients with AIDS had a higher SP-D than patients with higher CD4 counts. MBL levels in BAL were very low, and correlated with serum levels of MBL, which were not altered by HIV status or clinical AIDS.

Previous studies have shown that SP-D deficiency is associated with increased respiratory infection[7] and is associated with increased airway inflammation[5]. There was no evidence in this study to suggest that low SP-D level is a factor in the susceptibility of HIV infected patients to respiratory infection[21], or that lack of SP-D contributes to the pulmonary inflammation that is a feature of AIDS[22,23]. SP-D has been shown to bind to HIV envelope protein gp120 and inhibit HIV replication[3] so the increased SP-D levels seen in our subjects with AIDS may be an appropriate response, although we were not able to demonstrate a relationship between SP-D level and BAL HIV viral load. SP-D suppresses lymphocyte function by inhibiting T lymphocyte proliferation [24] therefore a decrease in CD4 count might be secondary to an increase in SP-D levels but we did not detect this association. Our patients were healthy and had normal chest radiographs but the increased SP-D seen in AIDS patients in this study is consistent with recent observations made of intensive care patients with pneumocytsis pneumonia in Germany[25].

MBL is a serum collectin that is found in lung at very low levels except in conditions of severe inflammation such as those that accompany pneumonia[11]. MBL leaks into the alveolar space in conditions of inflammation but levels do not increase as an acute phase protein[26]. We found that MBL levels were detectable but were not raised in BAL from HIV infected subjects or in subjects with AIDS, suggesting that the vascular integrity of the lung in these subjects was intact. We were able to correlate the MBL levels found in BAL with those in serum, both for HIV infected and normal subjects. We found no difference in MBL levels when we compared serum from normal subjects, HIV infected and AIDS patients. This differs from a study which showed a higher level of MBL in HIV infected patients[27] but is consistent with studies that showed no association between MBL level and either HIV infection, disease progression or AIDS[16,17]. Like SP-D, MBL binds HIV viral gp120 and can activate complement but we did not detect any association of MBL level with increased HIV viral load or AIDS.

## Conclusion

There was no evidence that susceptibility to infection or inflammation in the lungs of AIDS patients was due to altered levels of the collectins SP-D or MBL.

## Competing interests

The author(s) declare that they have no competing interests.

## Authors' contributions

KCJ was involved in the study design of the study, laboratory measurements, data analysis, and manuscript writing. SBG recruited the patients, carried out bronchoscopy and lavage procedures, analysed the data and was involved in manuscript writing. NF was involved in patient recruitment, clinical follow-up and manuscript writing. EEZ supervised the study and was involved in manuscript writing. All authors read and approved the final manuscript.
